# Muscle Glycogen Phosphorylase and Its Functional Partners in Health and Disease

**DOI:** 10.3390/cells10040883

**Published:** 2021-04-13

**Authors:** Marta Migocka-Patrzałek, Magdalena Elias

**Affiliations:** Department of Animal Developmental Biology, Faculty of Biological Sciences, University of Wroclaw, 50-335 Wroclaw, Poland; magdalena.elias@uwr.edu.pl

**Keywords:** PYGM, muscle glycogen phosphorylase, functional protein partners, glycogenolysis, McArdle disease, cancer, schizophrenia

## Abstract

Glycogen phosphorylase (PG) is a key enzyme taking part in the first step of glycogenolysis. Muscle glycogen phosphorylase (PYGM) differs from other PG isoforms in expression pattern and biochemical properties. The main role of PYGM is providing sufficient energy for muscle contraction. However, it is expressed in tissues other than muscle, such as the brain, lymphoid tissues, and blood. PYGM is important not only in glycogen metabolism, but also in such diverse processes as the insulin and glucagon signaling pathway, insulin resistance, necroptosis, immune response, and phototransduction. PYGM is implicated in several pathological states, such as muscle glycogen phosphorylase deficiency (McArdle disease), schizophrenia, and cancer. Here we attempt to analyze the available data regarding the protein partners of PYGM to shed light on its possible interactions and functions. We also underline the potential for zebrafish to become a convenient and applicable model to study PYGM functions, especially because of its unique features that can complement data obtained from other approaches.

## 1. Introduction

The main energy substrate in animal tissues is glucose, which is stored in the liver and muscles in the form of glycogen, a polymer consisting of glucose molecules. The molecules are connected via α-1,4-glycosidic and α-1,6-glycosidic bonds. Glycogen phosphorylase (GP) breaks α-1,4-glycosidic bonds, and the glucose-1-phosphate (G1P) molecule is released in the first step of glycogenolysis [1]. GP exists in three isoforms in the human body: phosphorylase, glycogen, liver (PYGL, UniProtKB no. P06737); phosphorylase, glycogen, brain (PYGB, no. P11216); and phosphorylase, glycogen, muscle (PYGM, no. P11217, commonly known as a muscle glycogen phosphorylase or myophosphorylase). The three isoforms differ in their physiological role and regulatory properties depending on the tissue in which they occur [2]. The PYGL, PYGB, and PYGM isoforms encoded by three distinct genes (on human chromosome 14q22, 20, and 11q13 respectively) share about 80% sequence identity at the protein level, and are structurally similar [3,4]. GP is a highly specialized enzyme, and its gene sequence is evolutionarily conserved. The comparative sequence analysis of phosphorylases shows that mammalian muscle and brain isoforms are more closely related to each other than to the liver form [5].

The metabolism, including glucose homeostasis, is linked to the circadian clock. The glycogen synthase (GS), glycogen phosphorylase, and glycogen level itself undergo regular changes in 24-h manner [6,7,8]. Despite that the GS and GP has an opposite functions, namely synthesis and sequestering of glycogen, their mRNA level is rising simultaneously at the morning phase in *Neurospora crassa*. The deletion of GP abolished the rhythmic GS gene expression, and glycogen accumulation. Regulation of GS and GP activity relies probably on allosteric changes and reversible phosphorylation. The GP is activated by phosphorylation, whereas phosphorylated GS is inactivated. This may result in the “switch like” system, in which one enzyme is active while the second is inactive [8]. The crucial role of clock-controlled glucose homeostasis and energy balance among mammals indicates that this mechanism may be evolutionarily conserved [6,7,9].

Additionally to their primary role in the first step of glycogenolysis, the GP isoforms also play a specific role in particular processes. The main function of PYGM and PYGB is their participation in adenosine triphosphate (ATP) production, gained from the glycogen deposits, to provide sufficient energy for biological processes in cells, such as contraction in the case of muscle. PYGL produces glucose molecules to maintain the glucose level in the bloodstream [5,10,11,12].

There are two mechanisms of GP activation: reversible phosphorylation and allosteric regulation. Allosteric regulation is understood as the balance between the T (tense, inactive) and R (relaxed, active) states, based on the conformational changes caused by the binding of regulatory molecules. The activators include adenosine monophosphate (AMP), inorganic phosphate (Pi), G1P, and glycogen, whereas inhibitors include ATP, glucose-6-phosphate (G6P), glucose, and purine [13]. PYGB and PYGM can be regulated by both serine phosphorylation and allosteric changes, but PYGL can only be regulated by reversible phosphorylation at serine 15 [4]. The difference between the biological role of PYGM and PYGB is based on their biochemical properties, particularly on the different affinity to AMP and glucose. When the level of AMP in the cell is low, PYGB reduces its enzymatic activity and does not respond to extracellular activation signals coming from the phosphorylation cascade. Therefore PYGB, present in fetal, brain, and heart tissue, is responsible for maintaining the optimal glycogen level for internal use. Indeed PYGB is responsible for providing an emergency energy source during periods of hypoxia, hypoglycemia, and ischemia [14,15]. On the other hand, PYGM is very active and responds to extracellular control via phosphorylation regardless of the cellular level of AMP. The mechanism of action results from its physiological role, which is a muscle contraction in response to neural and hormonal signals [16].

PYGM gains attention because of its crucial role in muscle functions and myopathies. Moreover, PYGM was found to be involved not only in glycogenolysis, but is also important in other physiological and pathological processes.

## 2. PYGM Expression in Different Tissues and Organs

*PYGM* mRNA expression analysis, based on transcriptomics datasets, shows that besides human skeletal muscles, it is also present in other tissues and organs. *PYGM* mRNA is present in organs containing skeletal muscle tissue, such as the tongue, glands, and esophagus. However, it is also detected e.g., in different parts of the brain, lymphoid tissues (tonsil), blood (granulocytes), salivary glands, male reproductive system, and adipose tissue [17]. PYGM and PYGB were shown to be colocalized in cardiomyocytes. Moreover, the heart-to-brain ratio of PYGM and PYGB protein and mRNA is similar, indicating that coexistence of the isoforms in heart muscle cells must be important for cardiac functions [18]. The tissue data for RNA expression obtained within one of the approaches, FANTOM5, reveal *PYGM* mRNA expression also in the eye (retina) [19].

The integrating quantitative transcriptomics performed on the human tissues, together with microarray-based immunohistochemistry, show that PYGM protein is expressed in the skeletal muscle tissue at a high level. At the same time, this analysis reveals that PYGM is also detectable in the cerebellum [17]. The antibody staining data confirm this finding and indicate that PYGM is present in the granular and white matter cells of the cerebellum, although its level is assessed as low. Glycogen muscle phosphorylase is the main form of GP expressed in glial cells in the human nervous system, specifically in astrocytes [20,21,22]. PYGM, identified by mass spectrometry, is also found in T lymphocytes, where it plays an important role in their immunological functions [23,24,25,26]. PYGM was also detected in the rat kidney homogenates, where it was localized in the interstitial cells of the cortex and outer medulla [27]. Additionally, the research data confirm the FANTOM5 results, showing that PYGM is expressed in the retinal pigment epithelium and cone photoreceptors [28,29].

The fact that PYGM is present not only in skeletal muscles, but also in several other tissues and organs, is probably due to its specific functions in these locations, probably connected with its biochemical properties.

## 3. The Biological Importance of PYGM

### 3.1. The Role of PYGM in Physiology

Muscle glycogen phosphorylase catalyzes the first step of glycogenolysis to meet the energy requirements for muscle activity. At the resting state, the inactive enzyme can be activated by AMP or inosine 5′-monophosphate (IMP), and is inhibited by ATP, G1P, and other metabolites. The coenzymes important for PYGM enzymatic activity regulation are pyridoxal phosphate (PLP, the active form of vitamin B6) [30], and Ras-related C3 botulinum toxin substrate 1 (Rac1) [23].

The datasets provided by Kyoto Encyclopedia of Genes and Genomes (KEGG), the biological pathways database, confirm that PYGM is involved mainly in the starch and sucrose metabolism and metabolic pathways, but indicates its involvement also in the insulin and glucagon signaling pathway, insulin resistance, and necroptosis [31].

**Figure 1:**
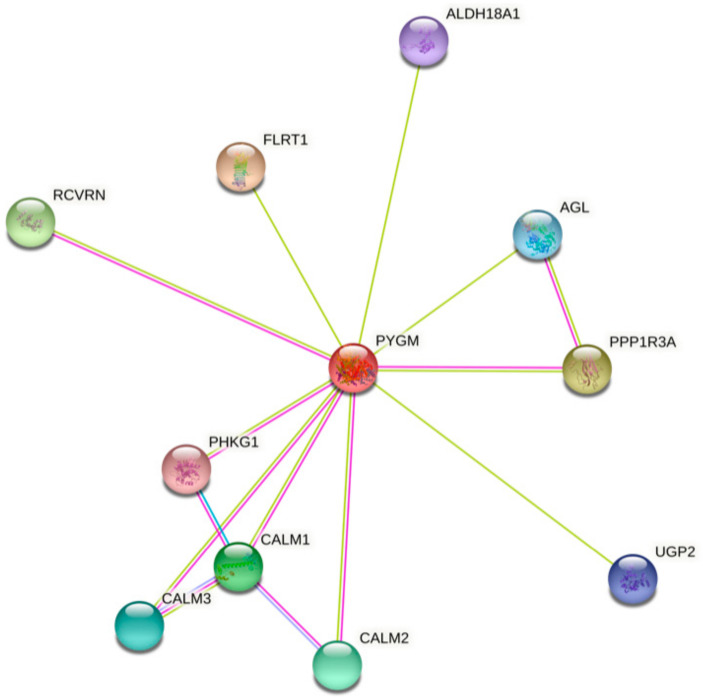
The human muscle glycogen phosphorylase (PYGM) protein–protein interaction network. The prediction, based on text mining, experiments, and databases, of possible PYGM associations. The edges indicate that the proteins are part of a physical complex. Four differently colored lines represent four types of evidence. A pink line indicates the experimentally determined interactions; light blue—database evidence; green—text mining evidence; dark blue—gene co-occurrence. ALDH18A1—aldehyde dehydrogenase 18 family member A1; AGL—amylo-alpha-1,6-glucosidase, 4-alpha-glucanotransferase; CALM1, 2, 3—calmodulin 1, 2, 3; FLRT1—fibronectin leucine rich transmembrane protein 1; PHKG1—phosphorylase kinase catalytic subunit gamma 1; PPP1R3A—protein phosphatase 1 regulatory subunit 3A; RCVRN—recoverin; UGP2—UDP-glucose pyrophosphorylase 2. According to the Protein–Protein Interaction Networks Functional Enrichment Analysis, STRING (accessed on 4 February 2021) [32].

The analysis of bioinformatics resources using the STRING tool shows additional possible protein–protein interactions, which may be important for the PYGM functions (Figure 1) [32]. The predicted physical network of functional protein partners with PYGM includes proteins involved in glycogen metabolism, specifically in glycogen breakdown (glycogenolysis), such as phosphorylase b kinase (PHK) catalytic subunit (PHKG1), and its delta subunit—calmodulin (encoded by three genes, *CALM1*, *CALM2*, and *CALM3*). The analysis additionally indicates the interaction with glycogen debranching enzyme (amylo-alpha-1,6-glucosidase, AGL) via protein phosphatase 1 (PPP1). Some of the predicted interactions have been experimentally verified. The two-hybrid experiment shows the interaction between PYGM and PPP1R3B (protein phosphatase 1, regulatory subunit 3B) [33].

PYGM plays a role in insulin and glucagon signaling, and insulin resistance pathways involving regulation of the glycogen level. PYGM participates in these processes through PHK and CALM in the signaling, and through PPP1 in the insulin resistance pathways [31,32]. The kinase PHK mediates the neural and hormonal regulation of glycogen breakdown by phosphorylating and thereby activating muscle glycogen phosphorylase. The phosphatase PP1 participates e.g., in the regulation of glycogen metabolism, muscle contraction, and protein synthesis. AGL is a multifunctional enzyme acting as a glycosyltransferase and glucosidase in glycogen debranching. CALM1 mediates the control of a large number of enzymes, ion channels, aquaporins, and other proteins through calcium binding [34].

PYGM is also involved in the phototransduction pathway, the process in which the photoreceptor cells generate electrical signals in response to captured photons. Probably PYGM is involved in the inactivation, recovery, and/or regulation of the phototransduction cascade through interaction with recoverin (RCVRN) and CALM1, both connected with Ca^2+^ cellular level regulation. The RCVRN, a low-molecular-weight, neuronal calcium sensor, is involved in phototransduction cascade regulation and signal transmission in a calcium-dependent manner [32,35]. So far, no experimental data explain the exact role of PYGM in this process. However, it is known that retinopathy can be one of the symptoms in muscle glycogen phosphorylase deficiency (McArdle disease) [29,36,37,38]. Analysis of the PYGM expression pattern leads to the conclusion that impaired glycogen metabolism, both in the retinal pigment epithelium and in cone photoreceptors, is involved in McArdle disease-linked retinopathy [29].

The role of PYGM in necroptosis described in the KEGG database, a type of programmed cell death with necrotic morphology, is based on the interaction with receptor interacting serine/threonine kinase (RIPK). RIPK3 activates glycogen phosphorylase and therefore influences glycogenolysis [39,40].

PYGM was also shown to play an important role in regulating the immune function of T cells. The stimulation of T cells with interleukin 2 (IL-2) leads to the activation of a small GTPase of the RAS family, RAC1. In its active configuration, RAC1 binds to PYGM and modulates PYGM enzymatic activity, leading to T-cell migration and proliferation [23,25,26]. Llavero et al. (2019) propose an additional possible mechanism of this signal cascade. Their model assumes that the PYGM activation (through RAC1) may be controlled by the epidermal growth factor receptor (EGFR) [41].

The PYGM protein–protein interaction network and its involvement in the biological processes are probably much wider, i.e., the possibly conserved role of glycolysis in promoting myoblast fusion-based muscle growth [37]. The formation of syncytial muscles is probably founded on glycolysis-based high-rate biomass production. Indeed the attenuation of one of the genes involved in glycolysis, phosphoglycerate mutase 2 (*Pglym78/pgam2*), leads to the formation of thinner muscles in *Drosophila melanogaster* embryos [42]. The Pygm protein level was shown to increase during zebrafish (*Danio rerio*) development, which correlates with the decrease in glycogen level. At the same time, the Pygm distribution in zebrafish muscles changed from dispersed to highly organized. These events correspond to increased energy demand, due to the first movements of the developing embryo [43].

The assembly performed within the Biological General Repository for Interaction Datasets (BioGRID) public database revealed almost 50 proteins involved in the biological interactions with PYGM (see Table S1) [44]. Therefore, it is highly probable that PYGM is an important factor involved not only in glycogenolysis but also in a diverse range of other physiological and pathological biological processes.

### 3.2. The Role of PYGM in Pathological Processes

#### 3.2.1. Muscle Glycogen Phosphorylase Deficiency (McArdle Disease)

Muscle glycogen phosphorylase deficiency (glycogen storage disorder type V called also McArdle disease; # 232600 in the Online Mendelian Inheritance in Man, OMIM, database) is the most common disorder of the skeletal muscle carbohydrate [45]. McArdle disease is an autosomal recessive metabolic disorder, caused by a lack of muscle glycogen phosphorylase. The most frequent mutations leading to McArdle disease are p.R50X, p.G205S, and L542T. So far, 206 mutations in the *PYGM* gene leading to McArdle disease development have been described. These mutations affect the processing of *PYGM* mRNA, may cause the absence of enzymatic activity, disrupt the interaction between enzyme dimers, or cause the lack of substrate binding [45,46,47]. The severity of the disease is most probably connected with diverse mechanisms, including post-transcriptional events, epigenetics factors, or modification of protein function [48]. The lack of active enzyme leads to the inability to gain the energy from glycogen needed for skeletal muscle contraction. Patients suffer from the onset of exercise intolerance and muscle cramps. Myoglobinuria may occur after physical effort, due to rhabdomyolysis. In some cases severe myoglobinuria may lead to acute renal failure [4,45].

Several case reports and longitudinal case studies have confirmed that retinopathy is an additional clinical phenotype feature associated with McArdle disease [29,36,37,38,49]. In the case of McArdle patients, the lack of PYGM may impair the ability of retinal pigment epithelium and cone photoreceptors to obtain sufficient energy, which may further lead to pathological changes [29]. Human retinal pigment epithelium cells express both brain and muscle forms of glycogen phosphorylase [28]. However, the presence of PYGB in epithelial cells may not be sufficient for efficient energy metabolism.

#### 3.2.2. PYGM in Schizophrenia

Disturbances in glutamate-mediated neurotransmission and alteration in energy metabolism in the dorsolateral prefrontal cortex (DLPFC) are observed in schizophrenia [22,50,51]. The glycogenolysis in neurons provides lactate as a transient energy supply. This source of energy is necessary for integrating the glutamatergic neurotransmission and glucose utilization processes. This mechanism could be altered in the disease and leads to an energy deficit. Pinacho et al. (2016) found that indeed the protein levels of PYGM and RAC1, a kinase that regulates PYGM activity, are reduced in the astrocytes in schizophrenia [22]. The interaction between PYGM and RAC1 in astrocytes may be similar to that described in the T cells [23,25,26]. The metabolic pathway in astrocytes, involving PYGM, could contribute to a transient local energy deficit in DLPFC in schizophrenia [22].

The equilibrium between brain and muscle isoform of glycogen phosphorylase in astrocytes may be controlled in a sex-dependent manner. It is because of the distinct astrocyte receptor profiles in males and females. The noradrenergic control over the astrocyte glycogen mobilization differs in the case of the adrenergic versus estrogen receptors. The exact mechanism needs further investigation, however authors conclude that glycogen turnover in the ventromedial hypothalamic nucleus, key structure responsible for the glucostatic control, is crucially important for maintaining brain functions. Therefore the future understanding of mechanism of noradrenergic control of glycogen level is an important issue [52]. Disturbances in glutamate-mediated neurotransmission have been observed not only in schizophrenia but also in various other neuropsychiatric disorders, including substance abuse, mood disorders, autism-spectrum disorders, and Alzheimer’s disease [53]. Therefore the regulation of glycogenolysis may be an important factor in the treatment of neuropsychiatric diseases. Especially in regards to identify the potential therapeutic targets for neuro-protective stabilization of glycogen level in systemic glucose dysregulation states.

The relationship between McArdle disease and schizophrenia, if any, is so far elusive. However, the recent data coming from the European registry for patients with McArdle disease and other muscle glycogenoses (EUROMAC) report the mental disorders reaching 6.6% (16 in the cohort of 241 patients). One case of schizophrenia was indicated within this category [54].

#### 3.2.3. PYGM in Cancer

**Figure 2:**
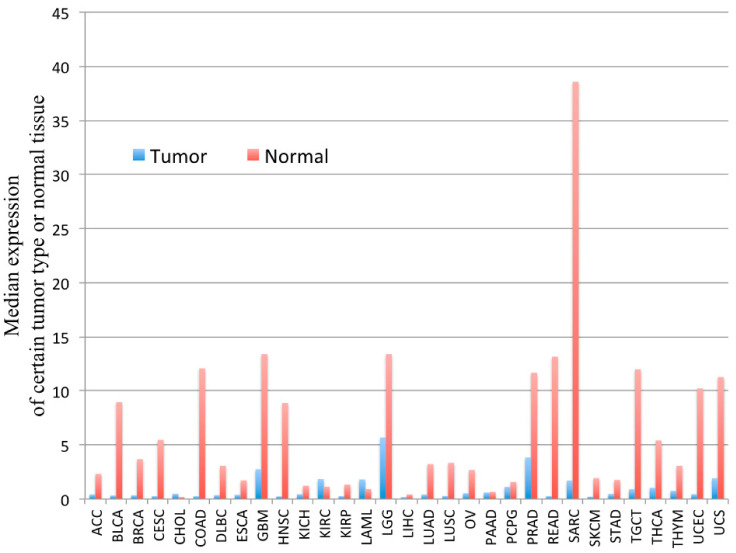
The *PYGM* gene expression profile across tumor samples and corresponding normal tissues. The height of the bar represents the median expression of a certain tumor type (blue) or normal tissue (red). ACC—adrenocortical carcinoma, BLCA—bladder urothelial carcinoma, BRCA—breast invasive carcinoma, CESC—cervical squamous cell carcinoma and endocervical adenocarcinoma, CHOL—cholangiocarcinoma, COAD—colon adenocarcinoma, DLBC—lymphoid neoplasm diffuse large b-cell lymphoma, ESCA—esophageal carcinoma, GBM—glioblastoma multiforme, HNSC—head and neck squamous cell carcinoma, KICH—kidney chromophobe, KIRC—kidney renal clear cell carcinoma, KIRP—kidney renal papillary cell carcinoma, LAML—acute myeloid leukemia, LGG—brain lower grade glioma, LIHC—liver hepatocellular carcinoma, LUAD—lung adenocarcinoma, LUSC—lung squamous cell carcinoma, MESO—mesothelioma, OV—ovarian serous cystadenocarcinoma, PAAD—pancreatic adenocarcinoma, PCPG—pheochromocytoma and paraganglioma, PRAD—prostate adenocarcinoma, READ—rectum adenocarcinoma, SARC—sarcoma, SKCM—skin cutaneous melanoma, STAD—stomach adenocarcinoma, TGCT—testicular germ cell tumors, THCA—thyroid carcinoma, THYM—thymoma, UCEC—uterine corpus endometrial carcinoma, UCS—uterine carcinosarcoma, UVM—uveal melanoma. According to the Gene Expression Profiling Interactive Analysis, GEPIA, on-line tool www.gepia.cancer (accessed on 4 February 2021) [55].

**Figure 3:**
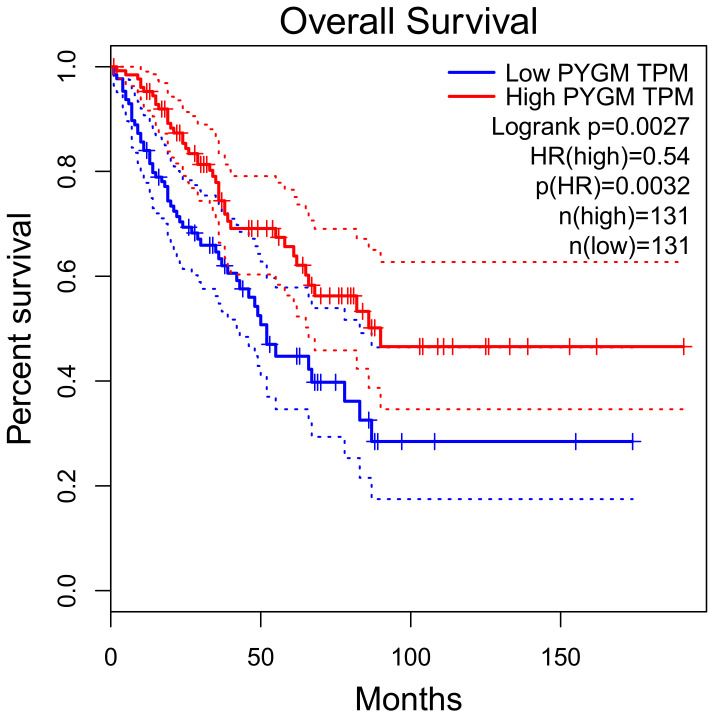
Overall survival rate of patients with sarcoma, depending on the PYGM gene expression rate. Low (median) PYGM expression correlated with poorer survival. Normalized RNA-sequencing data as transcripts per million (TPM). HR—hazard ratio calculated using Cox PH Model. The solid line represents the survival curve and the dotted line represents the 95% confidence interval. According to the Gene Expression Profiling Interactive Analysis, GEPIA, available online www.gepia.cancer (accessed on 4 February 2021) [55].

*PYGM* expression is down-regulated in cancer. According to the Gene Expression Profiling Interactive Analysis (GEPIA) of the RNA sequencing data, *PYGM* expression is lower in many types of cancer than in normal tissues (Figure 2). One of the largest differences is observed in the case of sarcoma (SARC), where *PYGM* expression is almost 20 times lower than in normal tissue. The analysis of data obtained from patients with SARC shows a significant difference in the survival rate between patients with low and high *PYGM* expression levels (Figure 3), indicating that *PYGM* may be a biomarker for SARC, and a useful parameter for disease prognosis [55]. Another example is the bioinformatics-based discovery indicating that *PYGM* and troponin C2, fast skeletal type (*TNNC2*), are significantly down-regulated in head and neck squamous cell carcinoma. The bioinformatics analysis of RNA sequencing data, confirmed experimentally, shows that both *PYGM* and *TNNC2* could be potentially used as therapeutics or biomarkers for diagnosis and prognosis in this type of cancer [56]. Interestingly, the expression of *TNNC1* and *TNNC2* genes was also shown to be significantly down-regulated in the case of PYGM deficiency (McArdle) disease [57].

Next-generation sequencing applied to three different subtypes of rare aggressive breast cancers (metaplastic, micropapillary, and pleomorphic lobular breast cancer) showed a 30% mutation rate in the *PYGM* gene. The missense mutation, similar in location to those identified in McArdle disease, probably leads to a loss-of-function effect, which could be one of the pathological mechanisms of cancer development. Immunohistochemical analysis confirmed lower PYGM expression in the tumor area when compared to non-malignant tissue surrounding tumor cells [58].

Multiple endocrine neoplasia type 1 (MEN1) is a cancer syndrome, inherited as an autosomal dominant trait with high penetrance. MEN1 patients suffer from the development of a variety of tumors, such as parathyroids, endocrine pancreas, and anterior pituitary [59,60]. Interestingly, the *MEN1* gene was found to be tightly linked to *PYGM* on the 11q13 chromosome. *MEN1* is located less than 100 kb telomeric to *PYGM* [61]. The correlation between *MEN1* and *PYGM* is worth noting, because the losses of heterozygosity regarding the 11q13 chromosome are often observed not only in *MEN1* but also in the case of sporadic carcinoid tumors of the lung and invasive breast cancers [62,63,64,65,66]. It is also known that the 11q13 chromosome rearrangements play an important role in B-cell non-Hodgkin’s lymphoma (B-NHL) [67].

On the other hand, Pastor et al. (2013) observed up-regulation of the PYGM protein in samples obtained from patients with lung cancer. The results revealed that PYGM protein and other proteins involved in the regulation of glycolysis, such as transketolase (TKT), fructose-bisphosphatase 1 (FBP1), aldolase fructose-bisphosphate A (ALDOA), and pyruvate kinase M1/2 (PKM2), were up-regulated in that set of experiments [68]. There are several possible explanations why the results differ from those previously reported, such as specificity of the patient group (15 males, mainly smokers), or a particular type of cancer with a distinct molecular mechanism [68].

In summary, the RNA sequencing results from public databases as well as the experimental data from different research indicates that PYGM could be an important factor in cancer development and progression. In the group of 241 McArdle disease and other muscle glycogenoses patients, the 4.6% cases of cancer were identified [54]. The glycogen metabolism plays a key role in tumorigenesis. Not only PYGM, but also PYGB level is up-regulated in different kinds of cancer such as colorectal cancer [69], hepatocellular carcinoma [70], prostate cancer [71], non-small cell lung cancer (NSCLC) [72], and ovarian cancer [73]. However, one should keep in mind that carcinogenesis is a complex process in which many factors interact together [74]. Therefore, the exact role of PYGM and other GPs in this process needs further study.

## 4. Why Use Zebrafish to Study PYGM?

Due to the many difficulties in studying biological processes using invasive techniques in humans, animal models have been utilized for decades for a better understanding of the molecular pathways, thus contributing to the progress in biological and medical sciences. Experiments performed with the use of animals also contributed significantly to the fundamental understanding of processes underlying human diseases. Research on animal models of human diseases has led to the development of effective therapies and treatment in many cases. Animal models are also widely used to perform research on glycogen storage diseases, a group of disorders connected with a defect in gene expression of specific enzymes involved in glycogen breakdown or synthesis, such as glycogen phosphorylase [45,47].

The muscle isoform is evolving at the slowest rate, showing great evolutionary conservation [5]. This phenomenon probably contributes to effective creation of animal research models of the disease caused by PYGM deficiency, McArdle disease [45]. Mice with PYGM deficiency were obtained by the introduction of nonsense p.R50X mutation into the *Pygm* gene, resulting in premature termination of translation. The mice display similar symptoms to those observed in McArdle patients, such as glycogen accumulation in muscle tissue, poor exercise performance, and a significantly elevated creatine kinase level in the blood [47]. The data obtained using the mouse model provide valuable knowledge regarding glycogen metabolism and McArdle disease. However, the generated mouse model has some disadvantages, such as much higher glycogen accumulation in muscles than observed in patients, because of faster metabolic rates in mice. Additionally, the mouse McArdle model shows a high level of perinatal and post-weaning mortality [75]. It was also observed that the glucose metabolism pathways, which were activated to compensate for the lack of PYGM, are different in the mouse model and humans affected by McArdle disease [76,77].

The zebrafish also has a great potential to become a useful model in research regarding PYGM functions in physiological and pathological biological processes. There are two homologous genes encoding muscle glycogen phosphorylase: *pygma* and *pygmb*. However, both zebrafish genes and proteins show great similarity to the human ones (85.0% amino acid sequence identity and 76.1% nucleotide sequence identity). The research results show that morpholino knockdown of *pygma* and *pygmb* in zebrafish leads to similar symptoms to those observed in McArdle patients. The *pygma* and *pygmb* knockdown resulted in a reduced Pygm level in zebrafish morphants, which exhibited changes in morphology, such as altered, disintegrated muscle structure, and accumulation of glycogen granules in the subsarcolemmal region [43]. The symptoms also include a reduction of mobility and swimming speed (data not published).

The advantages of zebrafish include relatively low breeding costs, the production of a large number (100–200 eggs per week) of ex utero developing embryos, and a short life cycle (they reach maturity within 3 months). It should be underlined that the external development of embryos in combination with the transparent body at the early developmental stages is a unique and very useful feature allowing for in vivo microscopic observations of e.g., developing muscles. Zebrafish tissues and organs share many features with humans at the anatomical, physiological, and molecular level. Many biochemical pathways also share high similarity, which facilitates the interpretation of test results from zebrafish. It is also worth noting that zebrafish genes associated with human diseases share a high level of conservation (84%) [78,79]. Therefore the zebrafish model has been used successfully in the modeling of many human diseases, including genetic and metabolic disorders [80,81,82]. The zebrafish models are also effectively used in neurodegenerative diseases, such as schizophrenia, because of e.g., similar brain architecture to humans and complexity of psychological processes such as capability of cognitive processing and complex decision making [83]. Moreover, zebrafish is a valuable, widely used model to study cancer. Its benefits include high evolutionary conservation of cancer-related molecular pathways compared to humans [84]. The research utilizing zebrafish allows for xenotransplantation studies (introducing human cancer cells into zebrafish embryos, enabling direct evaluation of patient-derived tumor specimens in vivo), evaluation of in vivo drug responses and kinetics. Furthermore, the zebrafish has some distinctive advantages, such as ease of testing and high-throughput screening of potentially therapeutic substances and drugs. Drug testing in the zebrafish model of human disease is a cost- and time-saving process, which can be used in the first phase of a clinical trial [85]. In summary, the use of zebrafish models in biological research can contribute to increased reproducibility and reliability of laboratory data.

The zebrafish’s potential to become a convenient and applicable model to study PYGM functions is also supported by the fact that PYGM protein partners have their zebrafish orthologs (Table 1). As mentioned above, the PYGM protein partners implicated in glycogen metabolism in humans are PHK subunits (PHKG1 and CALM1-3), AGL, PPP1CA, and PPP1R3B. The ortholog of human *PHKG1* is the gene *phkg1b* (phosphorylase kinase, gamma 1b) located on chromosome 21 in zebrafish. Zebrafish Phkg1b shows similar serine/threonine protein kinase activity to human PHKG1, and plays an analogical, important biological role in glycogenolysis by phosphorylating and activating GP. A mutation in the *PHK* gene leads e.g., to glycogen storage disease VI and IX in humans [86]. However, the PHK subunit PhKG1 can also play an important role in other pathologies. The PhKG1 inhibitor shows anti-angiogenic properties in zebrafish and human endothelial cell in vitro angiogenesis models. The PhKG1 level is also up-regulated in several human tumors [87]. CALM1, CALM2, and CALM3 consist of a delta subunit of PHK, acting as a regulatory subunit. However, it is not known if this particular PHK subunit is associated with glycogen storage disease IX [87]. Calmodulin also takes part in the control of a large number of enzymes, ion channels, and other proteins by Ca^2+^ binding. In the zebrafish, all three human proteins have their orthologs, but they are encoded by two genes (Table 1) [88,89,90].

One of the most important protein partners of human PYGM is AGL. The zebrafish ortholog of human AGL is agla (amylo-alpha-1,6-glucosidase, 4-alpha-glucanotransferase a). Similar to the human protein, the zebrafish enzyme has 4-alpha-glucanotransferase and amylo-alpha-1,6-glucosidase activity and plays the same glycogen debranching enzymatic role [90,91]. Mutations in this gene are associated with glycogen storage disease III (IIIa/Cori, IIIb, IIIc, IIId), affecting the liver as well as skeletal and cardiac muscles [88,89,92] (Table 1).

Zebrafish also has orthologs of human PPP1R3A and PPP1R3B, the catalytic and regulatory subunits of PPP1 (Table 1). The prediction of zebrafish ppp1r3aa and ppp1r3ab protein function shows glycogen- and PPP1-binding activity. The activities are similar to human PPP1R3A, protein phosphatase 1 regulatory subunit, which binds to muscle glycogen with high affinity and enhances dephosphorylation of phosphatase substrates. Correspondingly, the zebrafish ortholog of human PPP1R3B, ppp1r3b, plays a regulatory role in the glycogen biosynthetic process.

Other protein partners of PYGM, important in other processes, e.g., RCVRN—a member of the recoverin family of neuronal calcium sensors—are expressed in epiphysis, retina, retinal outer nuclear layer, and the retinal photoreceptor layer in zebrafish (ZFIN, accessed on 4 February 2021). Examples of zebrafish orthologs of human proteins are shown in Table 1.

The zebrafish have some disadvantages, which have to be taken into consideration during the experiment planning. The obvious one is the fact that zebrafish is not a mammal. This may be relevant, especially in the modelling human diseases, and performing clinical trials of potential drugs. The differences include also i.e., the development, which is external in zebrafish, therefore the embryos does not have placenta. Instead, the embryos are protected by chorion, acellular envelope surrounding mature eggs. Therefore the additional techniques to remove chorion, prior the toxicological tests, should be applied [93]. The additional challenges in drug testing include such limitations as poor water-solubility of some chemicals. In such case they have to be injected into fish to gain sufficient exposure. In the case of well water-soluble compounds, the yield used in the experiment is usually high, due to the fact that embryos have to be literally immersed in the solution. The direct and precise relations between doses used in toxicological test utilizing zebrafish, to concentrations active in mammals are not possible yet. Especially because the small fish size makes the methods for measuring plasma level of absorbed substances difficult [85,94]. Therefore some zebrafish features such as external development, and small size can be advantage or disadvantage, depending of the experiment planned.

The animal models of human diseases very rarely, if ever, present all features of the particular human disorder. However, the synthesis of research results obtained using different animal models allows for more straightforward extrapolation of observations to humans, especially as some features of zebrafish provide a powerful tool to complement other approaches [95].

## 5. Summary and Perspectives

The analysis of available data indicates that PYGM is involved in several important biological processes, especially those demanding rapid supply of energy. There is no correlation between PYGM level in muscles and diet [96]. However, the PYGM enzyme is effectively activated by phosphorylation in response to extracellular neural or hormonal signals [16]. This unique feature PYGM may be the reason why this particular isoform is involved not only in glycogen metabolism but also in insulin and glucagon signaling, and the insulin resistance pathway. The PYGM mechanism of action, allowing for rapid energy supply, seems to be relevant also in the brain astrocytes. The low level of PYGM and RAC1 in the astrocytes of patients suffering from schizophrenia may lead to local energy deficiency and contribute to disease pathophysiology [22].

**Figure 4:**
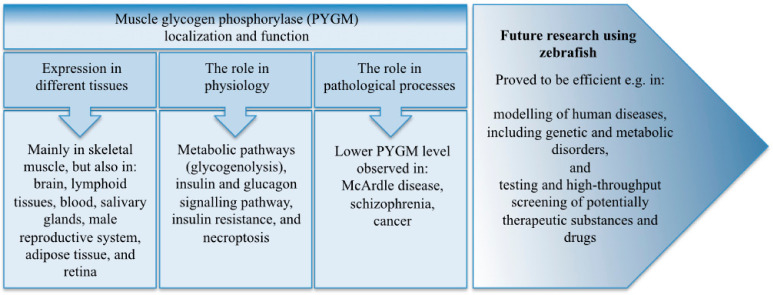
Muscle glycogen phosphorylase (PYGM) localization and function. The summary of the topics discussed in the review.

Glycogen metabolism, and the subsequent cellular energy balance, is important for all cells. It appears that this process also has a central role in cancer progression, since the PYGM level is highly down-regulated in many kinds of cancer, and decreased *PYGM* expression level correlates with patients’ poor survival rate. In contrast, glycogen synthase kinase 3 beta (GSK3b) is up-regulated in cancer, and the appropriate management of glycogen storage is important for cancer cell survival [58,97]. The proper amount of glycogen is also relevant in myoblast fusion-based muscle growth, since lower expression of genes connected with glycogenolysis, such as *Pygm* and *Pglym78/pgam2*, leads to thinner muscles in *D. melanogaster* and zebrafish [42,43] (Figure 4).

The data from research on human McArdle disease indicate that lack of PYGM leads to several genes’ down-regulation. That includes genes encoding acetyl-CoA carboxylase beta (*ACACB*), M-cadherin (*CADH15*), muscle creatine kinase (*CKMM*), calpain III (*CAPN3*), glycogen synthase (*GS*), and sarcoplasmic reticulum calcium ATPase 1 (*SERCA1*). Specifically, the GS and SERCA1 protein levels were reduced by 50% and 75%, respectively [57]. SERCA1 down-regulation may lead to impaired calcium transport in type II muscle fibers, responsible for isometric and intense dynamic exercises [57]. Also retinopathy, one of the symptoms of McArdle disease, is connected with Ca^2+^ level in the retinal pigment epithelium and cone photoreceptors, although the exact mechanism is unknown [29]. Additional experimental analysis is needed to gain knowledge regarding the potential role of PYGM in maintenance and/or regulation of the cellular level of Ca^2+^.

The analysis of research data from databases and publications shows several interesting connections between PYGM and other proteins, which need further investigation. For example, PYGM and TNNC2 are down-regulated in head and neck squamous cell carcinoma [56] and in McArdle patients [57].

Animals provide the possibility to perform research exploration. The comparison and synthesis of already known facts with new insights, obtained for example through zebrafish research, allows for the extrapolation of animal research data to humans. The research outcomes will also become a platform to compare the evolutionary similarities and/or differences between different species. The zebrafish is becoming a valid, useful pre-clinical model of human diseases, especially since preclinical drug trials on zebrafish are approved by the FDA (U.S. Food and Drug Administration).

## Figures and Tables

**Table 1:** Zebrafish (*Danio rerio*) orthologs of some human protein partners of muscle glycogen phosphorylase (PYGM).

Zebrafish (*Danio rerio*) Orthologs of Some Human (*Homo sapiens*) Protein Partners of Muscle Glycogen Phosphorylase (PYGM)			
Human		Zebrafish	
Protein	Systematic Name	Protein	Systematic Name
AGL	P35573	agla	A0A0R4IA63
UGP2	Q16851	ugp2a and ugp2b	B8JMZ1 and Q6NWJ8
PHK (PHKG1)	Q16816	phkg1b	Q503G9
PPP1R3A	Q16821	ppp1r3ab and ppp1r3aa	E7EZR5 and E7F487
PPP1R3B	Q86XI6	ppp1r3b	Q803M0
ALDH18A1	P54886	aldh18a1	A4IGC8
FLRT1	Q9NZU1	flrt1a	A8BBF0
CALM1	P0DP23	calm1a and calm1b	Q6PI52 and Q6PI52
CALM2	P0DP24	calm2a and calm2b	Q6PI52 and Q6PI52
CALM3	P0DP25	calm3a and calm3b	Q6PI52 and Q6PI52
RCVRN	P35243	rcvrn2	Q6PC38
RIPK1	Q13546	ripk1l	A8DZG7
RAC1	P63000	rac1a and rac1b	Q7ZSZ9 and Q29RC5

## Data Availability

Not applicable.

## Supplementary Materials

The following are available online at https://www.mdpi.com/article/10.3390/cells10040883/s1, Table S1: Assembly of phosphorylase, glycogen, muscle (PYGM) biological interactions.

## References

[1] Di Mauro S. (2007). Muscle glycogenoses: An overview. Acta Myol..

[2] Nogales-Gadea G., Santalla A., Brull A., De Luna N., Lucia A., Pinós T. (2015). The pathogenomics of McArdle disease—Genes, enzymes, models, and therapeutic implications. J. Inherit. Metab. Dis..

[3] Freeman S., Bartlett J.B., Convey G., Hardern I., Teague J.L., Loxham S.J.G., Allen J.M., Poucher S.M., Charles A.D. (2006). Sensitivity of glycogen phosphorylase isoforms to indole site inhibitors is markedly dependent on the activation state of the enzyme. Br. J. Pharmacol..

[4] Llavero F., Sastre A.A., Montoro M.L., Gálvez P., Lacerda H.M., Parada L.A., Zugaza J.L. (2019). McArdle Disease: New Insights into Its Underlying Molecular Mechanisms. Int. J. Mol. Sci..

[5] Hudson J.W., Golding G., Crerar M.M. (1993). Evolution of Allosteric Control in Glycogen Phosphorylase. J. Mol. Biol..

[6] Turek F.W., Joshu C., Kohsaka A., Lin E., Ivanova G., McDearmon E., Laposky A., Losee-Olson S., Easton A., Jensen D.R. (2005). Obesity and Metabolic Syndrome in Circadian Clock Mutant Mice. Science.

[7] Stenvers D.J., Scheer F.A.J.L., Schrauwen P., La Fleur S.E., Kalsbeek A. (2019). Circadian clocks and insulin resistance. Nat. Rev. Endocrinol..

[8] Baek M., Virgilio S., Lamb T.M., Ibarra O., Andrade J.M., Gonçalves R.D., Dovzhenok A., Lim S., Bell-Pedersen D., Bertolini M.C. (2019). Circadian clock regulation of the glycogen synthase (GSN) gene by WCC is critical for rhythmic glycogen metabolism inNeurospora crassa. Proc. Natl. Acad. Sci. USA.

[9] Scheer F.A.J.L., Hilton M.F., Mantzoros C.S., Shea S.A. (2009). Adverse metabolic and cardiovascular consequences of circadian misalignment. Proc. Natl. Acad. Sci. USA.

[10] Chasiotis D. (1983). The regulation of glycogen phosphorylase and glycogen breakdown in human skeletal muscle. Acta Physiol. Scand. Suppl..

[11] Hue L., Bontemps F., Hers H. (1975). The effects of glucose and of potassium ions on the interconversion of the two forms of glycogen phosphorylase and of glycogen synthetase in isolated rat liver preparations. Biochem. J..

[12] Ding Y.-J., Li G.-Y., Xu C.-D., Wu Y., Zhou Z.-S., Wang S.-G., Li C. (2020). Regulatory Functions of Nilaparvata lugens GSK-3 in Energy and Chitin Metabolism. Front. Physiol..

[13] Madsen N.B., Avramovic-Zikic O., Honikel K.O. (1973). Structure-Function Relationships in Glycogen Phosphorylase with Respect to Its Control Characteristics. Ann. N. Y. Acad. Sci..

[14] Lillpopp L., Tzikas S., Ojeda F., Zeller T., Baldus S., Bickel C., Sinning C.R., Wild P.S., Genth-Zotz S., Warnholtz A. (2012). Prognostic Information of Glycogen Phosphorylase Isoenzyme BB in Patients with Suspected Acute Coronary Syndrome. Am. J. Cardiol..

[15] Pudil R., Vašatová M., Lenco J., Tichy M., Řeháček V., Fucikova A., Horacek J.M., Vojacek J., Pleskot M., Stulik J. (2010). Plasma glycogen phosphorylase BB is associated with pulmonary artery wedge pressure and left ventricle mass index in patients with hypertrophic cardiomyopathy. Clin. Chem. Lab. Med..

[16] Crerar M.M., Karlsson O., Fletterick R.J., Hwang P.K. (1995). Chimeric Muscle and Brain Glycogen Phosphorylases Define Protein Domains Governing Isozyme-specific Responses to Allosteric Activation. J. Biol. Chem..

[17] Uhlén M., Fagerberg L., Hallström B.M., Lindskog C., Oksvold P., Mardinoglu A., Sivertsson Å., Kampf C., Sjöstedt E., Asplund A. (2015). Tissue-based map of the human proteome. Science.

[18] Schmid H., Pfeiffer-Guglielmi B., Dolderer B., Thiess U., Verleysdonk S., Hamprecht B. (2009). Expression of the Brain and Muscle Isoforms of Glycogen Phosphorylase in Rat Heart. Neurochem. Res..

[19] Abugessaisa I., Noguchi S., Hasegawa A., Harshbarger J., Kondo A., Lizio M., Severin J., Carninci P., Kawaji H., Kasukawa T. (2017). FANTOM5 CAGE profiles of human and mouse reprocessed for GRCh38 and GRCm38 genome assemblies. Sci. Data.

[20] Jakobsen E., Bak L.K., Walls A.B., Reuschlein A.-K., Schousboe A., Waagepetersen H.S. (2017). Glycogen Shunt Activity and Glycolytic Supercompensation in Astrocytes May Be Distinctly Mediated via the Muscle form of Glycogen Phosphorylase. Neurochem. Res..

[21] Pfeiffer-Guglielmi B., Fleckenstein B., Jung G., Hamprecht B. (2003). Immunocytochemical localization of glycogen phosphorylase isozymes in rat nervous tissues by using isozyme-specific antibodies. J. Neurochem..

[22] Pinacho R., Vila E., Prades R., Tarragó T., Castro E., Ferrer I., Ramos B. (2016). The glial phosphorylase of glycogen isoform is reduced in the dorsolateral prefrontal cortex in chronic schizophrenia. Schizophr. Res..

[23] Arrizabalaga O., Lacerda H.M., Zubiaga A.M., Zugaza J.L. (2012). Rac1 Protein Regulates Glycogen Phosphorylase Activation and Controls Interleukin (IL)-2-dependent T Cell Proliferation. J. Biol. Chem..

[24] De Luna N., Brull A., Lucia A., Santalla A., Garatachea N., Martí R., Andreu A.L., Pinós T. (2014). PYGM expression analysis in white blood cells: A complementary tool for diagnosing McArdle disease?. Neuromuscul. Disord..

[25] Llavero F., Urzelai B., Osinalde N., Gálvez P., Lacerda H.M., Parada L.A., Zugaza J.L. (2015). Guanine Nucleotide Exchange Factor αPIX Leads to Activation of the Rac 1 GTPase/Glycogen Phosphorylase Pathway in Interleukin (IL)-2-stimulated T Cells. J. Biol. Chem..

[26] Llavero F., Artaso A., Lacerda H.M., Parada L.A., Zugaza J.L. (2016). Lck/PLCγ control migration and proliferation of interleukin (IL)-2-stimulated T cells via the Rac1 GTPase/glycogen phosphorylase pathway. Cell. Signal..

[27] Schmid H., Dolderer B., Thiess U., Verleysdonk S., Hamprecht B. (2008). Renal Expression of the Brain and Muscle Isoforms of Glycogen Phosphorylase in Different Cell Types. Neurochem. Res..

[28] Hernández C., Garcia-Ramírez M., García-Rocha M., Saez-López C., Valverde Á.M., Guinovart J.J., Simó R. (2014). Glycogen storage in the human retinal pigment epithelium: A comparative study of diabetic and non-diabetic donors. Acta Diabetol..

[29] Vaclavik V., Naderi F., Schaller A., Escher P. (2020). Longitudinal case study and phenotypic multimodal characterization of McArdle disease-linked retinopathy: Insight into pathomechanisms. Ophthalmic Genet..

[30] Johnson L.N. (1992). Glycogen phosphorylase: Control by phosphorylation and allosteric effectors. FASEB J..

[31] Kanehisa M., Goto S., Furumichi M., Tanabe M., Hirakawa M. (2010). KEGG for representation and analysis of molecular networks involving diseases and drugs. Nucleic Acids Res..

[32] Szklarczyk D., Gable A.L., Lyon D., Junge A., Wyder S., Huerta-Cepas J., Simonovic M., Doncheva N.T., Morris J.H., Bork P. (2019). STRING v11: Protein–protein association networks with increased coverage, supporting functional discovery in genome-wide experimental datasets. Nucleic Acids Res..

[33] Blandin G., Marchand S., Charton K., Danièle N., Gicquel E., Boucheteil J.-B., Bentaib A., Barrault L., Stockholm D., Bartoli M. (2013). A human skeletal muscle interactome centered on proteins involved in muscular dystrophies: LGMD interactome. Skelet. Muscle.

[34] Adeva-Andany M.M., González-Lucán M., Donapetry-García C., Fernández-Fernández C., Ameneiros-Rodríguez E. (2016). Glycogen metabolism in humans. BBA Clin..

[35] Zang J., Neuhauss S.C.F. (2018). The Binding Properties and Physiological Functions of Recoverin. Front. Mol. Neurosci..

[36] Alsberge J.B., Chen J.J., Zaidi A.A., Fu A.D. (2018). Retinal Dystrophy in a Patient with Mcardle Disease. Retin. Cases Brief. Rep..

[37] Leonardy N.J., Harbin R.L., Sternberg P. (1988). Pattern Dystrophy of the Retinal Pigment Epithelium in a Patient with McArdle’s Disease. Am. J. Ophthalmol..

[38] Mahroo O.A., Khan K.N., Wright G., Ockrim Z., Scalco R.S., Robson A.G., Tufail A., Michaelides M., Quinlivan R., Webster A.R. (2019). Retinopathy Associated with Biallelic Mutations in PYGM (McArdle Disease). Ophthalmology.

[39] Liu Y., Liu T., Lei T., Zhang D., Du S., Girani L., Qi D., Lin C., Tong R., Wang Y. (2019). RIP1/RIP3-regulated necroptosis as a target for multifaceted disease therapy (Review). Int. J. Mol. Med..

[40] Zhang D.-W., Shao J., Lin J., Zhang N., Lu B.-J., Lin S.-C., Dong M.-Q., Han J. (2009). RIP3, an Energy Metabolism Regulator That Switches TNF-Induced Cell Death from Apoptosis to Necrosis. Science.

[41] Llavero F., Montoro M.L., Sastre A.A., Fernández-Moreno D., Lacerda H.M., Parada L.A., Lucia A., Zugaza J.L. (2019). Epidermal growth factor receptor controls glycogen phosphorylase in T cells through small GTPases of the RAS family. J. Biol. Chem..

[42] Tixier V., Bataillé L., Etard C., Jagla T., Weger M., Daponte J.P., Strähle U., Dickmeis T., Jagla K. (2013). Glycolysis supports embryonic muscle growth by promoting myoblast fusion. Proc. Natl. Acad. Sci. USA.

[43] Migocka-Patrzałek M., Lewicka A., Elias M., Daczewska M. (2020). The effect of muscle glycogen phosphorylase (Pygm) knockdown on zebrafish morphology. Int. J. Biochem. Cell Biol..

[44] Stark C., Breitkreutz B.J., Reguly T., Boucher L., Breitkreutz A., Tyers M. (2006). BioGRID: A general repository for interaction datasets. Nucleic Acids Res..

[45] Almodóvar-Payá A., Villarreal-Salazar M., De Luna N., Nogales-Gadea G., Real-Martínez A., Andreu A.L., Martín M.A., Arenas J., Lucia A., Vissing J. (2020). Preclinical Research in Glycogen Storage Diseases: A Comprehensive Review of Current Animal Models. Int. J. Mol. Sci..

[46] Nogales-Gadea G., Brull A., Santalla A., Andreu A.L., Arenas J., Martín M.A., Lucia A., De Luna N., Pinós T. (2015). McArdle Disease: Update of Reported Mutations and Polymorphisms in thePYGMGene. Hum. Mutat..

[47] Nogales-Gadea G., Pinós T., Lucia A., Arenas J., Cámara Y., Brull A., De Luna N., Martín M.A., Garcia-Arumí E., Marti R. (2012). Knock-in mice for the R50X mutation in the PYGM gene present with McArdle disease. Brain.

[48] Carvalho A.A.S., Christofolini D.M., Perez M.M., Alves B.C.A., Rodart I., Figueiredo F.W.S., Turke K.C., Feder D., Junior M.C.F., Nucci A.M. (2020). PYGM mRNA expression in McArdle disease: Demographic, clinical, morphological and genetic features. PLoS ONE.

[49] Casalino G., Chan W., McAvoy C., Coppola M., Bandello F., Bird A.C., Chakravarthy U. (2018). Multimodal imaging of posterior ocular involvement in McArdle’s disease. Clin. Exp. Optom..

[50] Sears S.M., Hewett S.J. (2021). Influence of glutamate and GABA transport on brain excitatory/inhibitory balance. Exp. Biol. Med..

[51] Uno Y., Coyle J.T. (2019). Glutamate hypothesis in schizophrenia. Psychiatry Clin. Neurosci..

[52] Briski K.P., Ibrahim M.M.H., Mahmood A.S.M.H., Alshamrani A.A. (2021). Norepinephrine Regulation of Ventromedial Hypothalamic Nucleus Astrocyte Glycogen Metabolism. Int. J. Mol. Sci..

[53] Moghaddam B., Javitt D.C. (2011). From Revolution to Evolution: The Glutamate Hypothesis of Schizophrenia and its Implication for Treatment. Neuropsychopharmacology.

[54] Scalco R.S., Lucia A., Santalla A., Martinuzzi A., Vavla M., Reni G., Toscano A., Musumeci O., Voermans N.C., EUROMAC Consortium (2020). Data from the European registry for patients with McArdle disease and other muscle glycogenoses (EUROMAC). Orphanet. J. Rare Dis..

[55] Tang Z., Li C., Kang B., Gao G., Li C., Zhang Z. (2017). GEPIA: A web server for cancer and normal gene expression profiling and interactive analyses. Nucleic Acids Res..

[56] Jin Y., Yang Y. (2019). Bioinformatics-based discovery of PYGM and TNNC2 as potential biomarkers of head and neck squamous cell carcinoma. Biosci. Rep..

[57] Nogales-Gadea G., Consuegra-García I., Rubio J.C., Arenas J., Cuadros M., Camara Y., Torres-Torronteras J., Fiuza-Luces C., Lucia A., Martín M.A. (2012). A Transcriptomic Approach to Search for Novel Phenotypic Regulators in McArdle Disease. PLoS ONE.

[58] Dieci M.V., Smutná V., Scott V., Yin G., Xu R., Vielh P., Mathieu M.-C., Vicier C., Laporte M., Drusch F. (2016). Whole exome sequencing of rare aggressive breast cancer histologies. Breast Cancer Res. Treat..

[59] Al-Salameh A., Baudry C., Cohen R. (2018). Update on multiple endocrine neoplasia Type 1 and 2. La Presse Médicale.

[60] Kedra D., Seroussi E., Fransson I., Trifunovic J., Clark M., Lagercrantz J., Blennow E., Mehlin H., Dumanski J. (1997). The germinal center kinase gene and a novel CDC25-like gene are located in the vicinity of the PYGM gene on 11q13. Qual. Life Res..

[61] Lemmes I., Van de Ven W.J.M., Kas K., Zhang C.X., Giraud S., Wautot V., Buisson N., Pugeat M., Peix J.L., Caldener A. (1998). The search for the MEN1 gene. The European Consortium on MEN-1. Intern. Med..

[62] Asteria C., Anagni M., Persani L., Beck-Peccoz P. (2001). Loss of heterozygosity of the MEN1 gene in a large series of TSH-secreting pituitary adenomas. J. Endocrinol. Investig..

[63] Bièche I., Lidereau R. (1995). Genetic alterations in breast cancer. Genes Chromosom. Cancer.

[64] Debelenko L.V., Brambilla E., Agarwal S.K., Swalwell J.I., Kester M.B., Lubensky I.A., Zhuang Z., Guru S.C., Manickam P., Olufemi S.-E. (1997). Identification of MEN1 gene mutations in sporadic carcinoid tumors of the lung. Hum. Mol. Genet..

[65] Petzmann S., Ullmann R., Klemen H., Renner H., Popper H.H. (2001). Loss of heterozygosity on chromosome arm 11q in lung carcinoids. Hum. Pathol..

[66] Zhuang Z., Merino M.J., Chuaqui R., Liotta L., Emmert-Buck M.R. (1995). Identical allelic loss on chromosome 11q13 in microdissected in situ and invasive human breast cancer. Cancer Res..

[67] Vandenberghe E., Peeters C.D.W., Wlodarska I., Stul M., Louwagie A., Verhoef G., Thomas J., Criel A., Cassiman J.J., Mecucci C. (1992). Chromosome 11q rearrangements in B non Hodgkin’s lymphoma. Br. J. Haematol..

[68] Pastor M., Nogal A., Molina-Pinelo S., Melendez R., Salinas A., De La Peña M.G., Martín-Juan J., Corral J., Garcia-Carbonero R., Carnero A. (2013). Identification of proteomic signatures associated with lung cancer and COPD. J. Proteom..

[69] Tashima S., Shimada S., Yamaguchi K., Tsuruta J., Ogawa M. (2000). Expression of brain-type glycogen phosphorylase is a potentially novel early biomarker in the carcinogenesis of human colorectal carcinomas. Am. J. Gastroenterol..

[70] Cui G., Wang H., Liu W., Xing J., Song W., Zeng Z., Liu L., Wang H., Wang X., Luo H. (2020). Glycogen Phosphorylase B Is Regulated by miR101-3p and Promotes Hepatocellular Carcinoma Tumorigenesis. Front. Cell Dev. Biol..

[71] Wang Z., Han G., Liu Q., Zhang W., Wang J. (2018). Silencing of PYGB suppresses growth and promotes the apoptosis of prostate cancer cells via the NF-κB/Nrf2 signaling pathway. Mol. Med. Rep..

[72] Lee M.K., Kim J.H., Lee C.H., Kim J.M., Kang C.D., Kim Y.D., Choi K.U., Kim H.W., Kim J.Y., Park D.Y. (2006). Clinicopathological significance of BGP expression in non-small-cell lung carcinoma: Relationship with histological type, microvessel density and patients’ survival. Pathology.

[73] Zhou Y., Jin Z., Wang C. (2019). Glycogen phosphorylase B promotes ovarian cancer progression via Wnt/β-catenin signaling and is regulated by miR-133a-3p. Biomed. Pharmacother..

[74] Davis A., Gao R., Navin N. (2017). Tumor evolution: Linear, branching, neutral or punctuated?. Biochim. Biophys. Acta (BBA) Bioenerg..

[75] Real-Martinez A., Brull A., Huerta J., Tarrasó G., Lucia A., Martin M.A., Arenas J., Andreu A.L., Nogales-Gadea G., Vissing J. (2019). Low survival rate and muscle fiber-dependent aging effects in the McArdle disease mouse model. Sci. Rep..

[76] Krag T.O., Pinós T., Nielsen T.L., Duran J., Garcia-Rocha M., Andreu A.L., Vissing J. (2016). Differential glucose metabolism in mice and humans affected by McArdle disease. Am. J. Physiol. Integr. Comp. Physiol..

[77] Nielsen T.L., Pinós T., Brull A., Vissing J., Krag T.O. (2018). Exercising with blocked muscle glycogenolysis: Adaptation in the McArdle mouse. Mol. Genet. Metab..

[78] Dubińska-Magiera M., Daczewska M., Lewicka A., Migocka-Patrzałek M., Niedbalska-Tarnowska J., Jagla K. (2016). Zebrafish: A Model for the Study of Toxicants Affecting Muscle Development and Function. Int. J. Mol. Sci..

[79] Plantié E., Migocka-Patrzałek M., Daczewska M., Jagla K. (2015). Model Organisms in the Fight against Muscular Dystrophy: Lessons from Drosophila and Zebrafish. Molecules.

[80] Benchoula K., Khatib A., Jaffar A., Ahmed Q.U., Sulaiman W.M.A.W., Wahab R.A., El-Seedi H.R. (2019). The promise of zebrafish as a model of metabolic syndrome. Exp. Anim..

[81] Bradford Y.M., Toro S., Ramachandran S., Ruzicka L., Howe D.G., Eagle A., Kalita P., Martin R., Moxon S.A.T., Schaper K. (2017). Zebrafish Models of Human Disease: Gaining Insight into Human Disease at ZFIN. ILAR J..

[82] Seth A., Stemple D.L., Barroso I. (2013). The emerging use of zebrafish to model metabolic disease. Dis. Model. Mech..

[83] Morris J.A. (2009). Zebrafish: A model system to examine the neurodevelopmental basis of schizophrenia. Prog. Brain Res..

[84] Hason M., Bartůněk P. (2019). Zebrafish Models of Cancer—New Insights on Modeling Human Cancer in a Non-Mammalian Vertebrate. Genes.

[85] Cassar S., Adatto I., Freeman J.L., Gamse J.T., Iturria I., Lawrence C., Muriana A., Peterson R.T., Van Cruchten S., Zon L.I. (2020). Use of Zebrafish in Drug Discovery Toxicology. Chem. Res. Toxicol..

[86] Kishnani P.S., Goldstein J., Austin S.L., Arn P., Bachrach B., Bali D.S., Chung W.K., El-Gharbawy A., Brown L.M., on behalf of the ACMG Work Group on Diagnosis and Management of Glycogen Storage Diseases Type VI and IX (2019). Diagnosis and management of glycogen storage diseases type VI and IX: A clinical practice resource of the American College of Medical Genetics and Genomics (ACMG). Genet. Med..

[87] Camus S., Quevedo C., Menéndez S., Paramonov I., Stouten P.F.W., Janssen R.A.J., Rueb S., He S., Snaar-Jagalska B., Laricchia-Robbio L. (2011). Identification of phosphorylase kinase as a novel therapeutic target through high-throughput screening for anti-angiogenesis compounds in zebrafish. Oncogene.

[88] NCBI. https://www.ncbi.nlm.nih.gov/gene.

[89] UniProt. https://www.uniprot.org/uniprot.

[90] ZFIN. http://zfin.org/.

[91] Quach H.N.B., Tao S., Vrljicak P., Joshi A., Ruan H., Sukumaran R., Varshney G.K., LaFave M.C., Burgess S.M., Winkler C. (2015). A Multifunctional Mutagenesis System for Analysis of Gene Function in Zebrafish. G3 Genes Genomes Genet..

[92] Dagli A., Sentner C.P., Weinstein D.A., Adam M.P., Ardinger H.H., Pagon R.A., Wallace S.E., Bean L.J., Mirzaa G., Amemiya A. (1993). Glycogen Storage Disease Type III. GeneReviews^®^.

[93] Henn K., Braunbeck T. (2011). Dechorionation as a tool to improve the fish embryo toxicity test (FET) with the zebrafish (Danio rerio). Comp. Biochem. Physiol. Part C Toxicol. Pharmacol..

[94] Grech A., Tebby C., Brochot C., Bois F.Y., Bado-Nilles A., Dorne J.-L., Quignot N., Beaudouin R. (2019). Generic physiologically-based toxicokinetic modelling for fish: Integration of environmental factors and species variability. Sci. Total Environ..

[95] De Abreu M.S., Kalueff A.V. (2021). Of mice and zebrafish: The impact of the experimenter identity on animal behavior. Lab. Anim..

[96] Stygar D., Andrare D., Bażanów B., Chełmecka E., Sawczyn T., Skrzep-Poloczek B., Olszańska E., Karcz K.W., Jochem J. (2019). The Impact of DJOS Surgery, a High Fat Diet and a Control Diet on the Enzymes of Glucose Metabolism in the Liver and Muscles of Sprague-Dawley Rats. Front. Physiol..

[97] Pelletier J., Bellot G., Gounon P., Lacas-Gervais S., Pouysségur J., Mazure N.M. (2012). Glycogen Synthesis is Induced in Hypoxia by the Hypoxia-Inducible Factor and Promotes Cancer Cell Survival. Front. Oncol..

