# Supplementary material for: Muscle Glycogen Phosphorylase and Its Functional Partners in Health and Disease
Source: Cells. 2021 Apr 13;10(4):883. doi: 10.3390/cells10040883 (PMC8070155; doi:10.3390/cells10040883)
Supplement: Supplementary file 1 [file cells-10-00883-s001.pdf]

Table S1. Assembly of phosphorylase, glycogen, muscle (PYGM) biological interactions. Performed within the framework of the Biological General Repository for Interaction Datasets (BioGRID) public database (thebiogrid.org, 20.02.2021) [1].

Phosphorylase, glycogen, muscle (PYGM) biological interactions					
No.	Interactor		Systematic name	Organism	Source
1	ABTB2	ankyrin repeat and BTB (POZ) domain containing 2	Q8N961	*H. sapiens*	[2]
2	AGRN	agrin	O00468	*H. sapiens*	[3]
3	AGTPBP1	ATP/GTP binding protein 1	Q9UPW5	*H. sapiens*	[4]
4	ARID1B	AT rich interactive domain 1B (SWI1-like)	Q8NFD5	*H. sapiens*	[4]
5	BRCA2	breast cancer 2, early onset	P51587	*H. sapiens*	[5]
6	CCDC15	coiled-coil domain containing 15	Q8C9M2	*M. musculus*	[6]
7	CDC42BPB	CDC42 binding protein kinase beta (DMPK-like)	Q9Y5S2	*H. sapiens*	[4]
8	CLASP2	cytoplasmic linker associated protein 2	O75122	*H. sapiens*	[4]
9	DEGS1	delta(4)-desaturase, sphingolipid 1	MIG15	*H. sapiens*	[7]
10	DNM2	dynamin 2	P50570	*H. sapiens*	[4]
11	FAM110A	family with sequence similarity 110, member A	Q9BQ89	*H. sapiens*	[4]
12	FANCD2	Fanconi anemia, complementation group D2	Q9BXW9	*H. sapiens*	[8]
13	GBAS	glioblastoma amplified sequence	O75323	*H. sapiens*	[4]
14	GBE1	glucan (1,4-alpha-), branching enzyme 1	Q04446	*H. sapiens*	[9]
15	IGBP1	immunoglobulin (CD79A) binding protein 1	P78318	*H. sapiens*	[4]
16	INTS4	integrator complex subunit 4	Q96HW7	*H. sapiens*	[4]
17	KRT17	keratin 17	Q04695	*H. sapiens*	[10]
18	LMNA	lamin A/C	P02545	*H. sapiens*	[4]
19	LZTR1	leucine-zipper-like	Q8N653	*H. sapiens*	[2]

		transcription regulator 1			
20	MCM2	minichromosome maintenance complex component 2	P49736	*H. sapiens*	[11]
21	MECOM	MDS1 and EVI1 complex locus	Q03112	*H. sapiens*	[2]
22	PACSIN3	protein kinase C and casein kinase substrate in neurons 3	Q9UKS6	*H. sapiens*	[7]
23	PDE4DIP	phosphodiesterase 4D interacting protein	Q5VU43	*H. sapiens*	[4]
24	PLEKHA4	pleckstrin homology domain containing, family A (phosphoinositide binding specific) member 4	Q9H4M7	*H. sapiens*	[12]
25	POMP	proteasome maturation protein	Q9Y244	*H. sapiens*	[4]
26	PPP1CA	protein phosphatase 1, catalytic subunit, alpha isozyme	P62136	*H. sapiens*	[13]
27	PPP1R3B	protein phosphatase 1, regulatory subunit 3B	Q86XI6	*H. sapiens*	[4]
28	PPP2CA	protein phosphatase 2, catalytic subunit, alpha isozyme	P67775	*H. sapiens*	[14]
29	PRKAB2	protein kinase, AMP-activated, beta 2 non-catalytic subunit	O43741	*H. sapiens*	[15]
30	PRKAB2	protein kinase, AMP-activated, beta 2 non-catalytic subunit	O43741	*H. sapiens*	[16]
31	PRKAB2	protein kinase, AMP-activated, beta 2 non-catalytic subunit	O43741	*H. sapiens*	[17]
32	PYGB	phosphorylase, glycogen; brain	P11216	*H. sapiens*	[16]
33	PYGL	phosphorylase, glycogen, liver	P06737	*H. sapiens*	[16]
34	S100A1	S100 calcium binding protein A1	P23297	*H. sapiens*	[18]
35	S100A1	S100 calcium binding protein A1	P23297	*H. sapiens*	[18]
36	SGCG	sarcoglycan, gamma (35kDa dystrophin-associated glycoprotein)	Q13326	*H. sapiens*	[4]
37	SRP72	signal recognition particle 72 kDa	O76094	*H. sapiens*	[4]

38	TLE3	transducin-like enhancer of split 3	Q04726	*H. sapiens*	[19]
39	TOP1	topoisomerase (DNA) I	P11387	*H. sapiens*	[20]
40	TRAPPC2	trafficking protein particle complex 2	P0DI81	*H. sapiens*	[4]
41	TRIM54	tripartite motif containing 54	Q9BYV2	*H. sapiens*	[4]
42	TRIM55	tripartite motif containing 55	Q9BYV6	*H. sapiens*	[4]
43	TRIM63	tripartite motif containing 63, E3 ubiquitin protein ligase	Q969Q1	*H. sapiens*	[21]
44	TTN	titin	Q8WZ42	*H. sapiens*	[4]
45	WDYHV1	WDYHV motif containing 1	Q96HA8	*H. sapiens*	[17]
46	WWP1	WW domain containing E3 ubiquitin protein ligase 1	Q9H0M0	*H. sapiens*	[1]

References

[1] Stark, C. BioGRID: A General Repository for Interaction Datasets. *Nucleic Acids Res.*, **2006**, *34* (90001), D535–D539. https://doi.org/10.1093/nar/gkj109.

[2] Huttlin, E. L.; Ting, L.; Bruckner, R. J.; Gebreab, F.; Gygi, M. P.; Szpyt, J.; Tam, S.; Zarraga, G.; Colby, G.; Baltier, K.; et al. The BioPlex Network: A Systematic Exploration of the Human Interactome. *Cell*, **2015**, *162* (2), 425–440. https://doi.org/10.1016/j.cell.2015.06.043.

[3] Rivera, C.; Zandonadi, F. S.; Sánchez-Romero, C.; Soares, C. D.; Granato, D. C.; González-Arriagada, W. A.; Paes Leme, A. F. Agrin Has a Pathological Role in the Progression of Oral Cancer. *Br. J. Cancer*, **2018**, *118* (12), 1628–1638. https://doi.org/10.1038/s41416-018-0135-5.

[4] Blandin, G.; Marchand, S.; Charton, K.; Danièle, N.; Gicquel, E.; Boucheteil, J.-B.; Bentaib, A.; Barrault, L.; Stockholm, D.; Bartoli, M.; et al. A Human Skeletal Muscle Interactome Centered on Proteins Involved in Muscular Dystrophies: LGMD Interactome. *Skelet. Muscle*, **2013**, *3* (1), 3. https://doi.org/10.1186/2044-5040-3-3.

[5] Malik, S.; Saito, H.; Takaoka, M.; Miki, Y.; Nakanishi, A. BRCA2 Mediates Centrosome Cohesion via an Interaction with Cytoplasmic Dynein. *Cell Cycle*, **2016**, *15* (16), 2145–2156. https://doi.org/10.1080/15384101.2016.1195531.

[6] Hutchins, J. R. A.; Toyoda, Y.; Hegemann, B.; Poser, I.; Heriche, J. K.; Sykora, M. M.; Augsburg, M.; Hudecz, O.; Buschhorn, B. A.; Bulkescher, J.; et al. Systematic Analysis of Human Protein Complexes Identifies Chromosome Segregation Proteins. *Science*, **2010**, *328* (5978), 593–599. https://doi.org/10.1126/science.1181348.

[7] Ewing, R. M.; Chu, P.; Elisma, F.; Li, H.; Taylor, P.; Climie, S.; McBroom‐Cerajewski, L.; Robinson, M. D.; O'Connor, L.; Li, M.; et al. Large‐scale Mapping of Human Protein–Protein Interactions by Mass Spectrometry. *Mol. Syst. Biol.*, **2007**, *3* (1), 89. https://doi.org/10.1038/msb4100134.

[8] Pladevall-Morera, D.; Munk, S.; Ingham, A.; Garribba, L.; Albers, E.; Liu, Y.; Olsen, J. V.; Lopez-Contreras, A. J. Proteomic Characterization of Chromosomal Common Fragile Site (CFS)-Associated Proteins Uncovers ATRX as a Regulator of CFS Stability. *Nucleic Acids Res.*, **2019**, *47* (15), 8004–8018. https://doi.org/10.1093/nar/gkz510.

[9] Wan, C.; Borgeson, B.; Phanse, S.; Tu, F.; Drew, K.; Clark, G.; Xiong, X.; Kagan, O.; Kwan, J.; Bezginov, A.; et al. Panorama of Ancient Metazoan Macromolecular Complexes. *Nature*, **2015**, *525* (7569), 339–344. https://doi.org/10.1038/nature14877.

[10] Yang, L.; Jin, L.; Ke, Y.; Fan, X.; Zhang, T.; Zhang, C.; Bian, H.; Wang, G. E3 Ligase Trim21 Ubiquitylates and Stabilizes Keratin 17 to Induce STAT3 Activation in Psoriasis. *J. Invest. Dermatol.*, **2018**, *138* (12), 2568–2577. https://doi.org/10.1016/j.jid.2018.05.016.

[11] Drissi, R.; Dubois, M.-L.; Douziech, M.; Boisvert, F.-M. Quantitative Proteomics Reveals Dynamic Interactions of the Minichromosome Maintenance Complex (MCM) in the Cellular Response to Etoposide Induced DNA Damage*. *Mol. Cell. Proteomics*, **2015**, *14* (7), 2002–2013. https://doi.org/10.1074/mcp.M115.048991.

[12] Shami Shah, A.; Batrouni, A. G.; Kim, D.; Punyala, A.; Cao, W.; Han, C.; Goldberg, M. L.; Smolka, M. B.; Baskin, J. M. PLEKHA4/Kramer Attenuates Dishevelled Ubiquitination to Modulate Wnt and Planar Cell Polarity Signaling. *Cell Rep.*, **2019**, *27* (7), 2157-2170.e8. https://doi.org/10.1016/j.celrep.2019.04.060.

[13] Skene-Arnold, T. D.; Luu, H. A.; Uhrig, R. G.; De Wever, V.; Nimick, M.; Maynes, J.; Fong, A.; James, M. N. G.; Trinkle-Mulcahy, L.; Moorhead, G. B.; et al. Molecular Mechanisms Underlying the Interaction of Protein Phosphatase-1c with ASPP Proteins. *Biochem. J.*, **2013**, *449* (3), 649–659. https://doi.org/10.1042/BJ20120506.

[14] Okamoto, K.; Li, H.; Jensen, M. R.; Zhang, T.; Taya, Y.; Thorgeirsson, S. S.; Prives, C. Cyclin G Recruits PP2A to Dephosphorylate Mdm2. *Mol. Cell*, **2002**, *9* (4), 761–771. https://doi.org/10.1016/S1097-2765(02)00504-X.

[15] Corominas, R.; Yang, X.; Lin, G. N.; Kang, S.; Shen, Y.; Ghamsari, L.; Broly, M.; Rodriguez, M.; Tam, S.; Trigg, S. A.; et al. Protein Interaction Network of Alternatively Spliced Isoforms from Brain Links Genetic Risk Factors for Autism. *Nat. Commun.*, **2014**, *5* (1), 3650. https://doi.org/10.1038/ncomms4650.

[16] Luck, K.; Kim, D.-K.; Lambourne, L.; Spirohn, K.; Begg, B. E.; Bian, W.; Brignall, R.; Cafarelli, T.; Campos-Laborie, F. J.; Charloteaux, B.; et al. A Reference Map of the Human Binary Protein Interactome. *Nature*, **2020**, *580* (7803), 402–408. https://doi.org/10.1038/s41586-020-2188-x.

[17] Rolland, T.; Taşan, M.; Charloteaux, B.; Pevzner, S. J.; Zhong, Q.; Sahni, N.; Yi, S.; Lemmens, I.; Fontanillo, C.; Mosca, R.; et al. A Proteome-Scale Map of the Human Interactome Network. *Cell*, **2014**, *159* (5), 1212–1226. https://doi.org/10.1016/j.cell.2014.10.050.

[18] Kiewitz, R.; Acklin, C.; Schäfer, B. W.; Maco, B.; Uhrík, B.; Wuytack, F.; Erne, P.; Heizmann, C. W. Ca2+-Dependent Interaction of S100A1 with the Sarcoplasmic Reticulum Ca2+-ATPase2a and Phospholamban in the Human Heart. *Biochem. Biophys. Res. Commun.*, **2003**, *306* (2), 550–557. https://doi.org/10.1016/S0006-291X(03)00987-2.

[19] Huttlin, E. L.; Bruckner, R. J.; Paulo, J. A.; Cannon, J. R.; Ting, L.; Baltier, K.; Colby, G.; Gebreab, F.; Gygi, M. P.; Parzen, H.; et al. Architecture of the Human Interactome Defines Protein Communities and Disease Networks. *Nature*, **2017**, *545* (7655), 505–509. https://doi.org/10.1038/nature22366.

[20] Trzcińska, A. M.; Girstun, A.; Piekiełko, A.; Kowalska-Loth, B.; Staroń, K. [No Title Found]. *Mol. Biol. Rep.*, **2002**, *29* (4), 347–352. https://doi.org/10.1023/A:1021237021338.

[21] Hirner, S.; Krohne, C.; Schuster, A.; Hoffmann, S.; Witt, S.; Erber, R.; Sticht, C.; Gasch, A.; Labeit, S.; Labeit, D. MuRF1-Dependent Regulation of Systemic Carbohydrate Metabolism as Revealed from Transgenic Mouse Studies. *J. Mol. Biol.*, **2008**, *379* (4), 666–677. https://doi.org/10.1016/j.jmb.2008.03.049.
